# Evaluation of Physicochemical Properties of Sustained-Release Membranes Based on Analytic Hierarchy

**DOI:** 10.3390/membranes13030313

**Published:** 2023-03-09

**Authors:** Haonan Sun, Tao Lei, Xianghong Guo, Jianxin Liu, Jiangjian Lv

**Affiliations:** 1 College of Water Resource Science and Engineering, Taiyuan University of Technology, Taiyuan 030024, China; 2 College of Chemical Engineering and Technology, Taiyuan University of Technology, Taiyuan 030024, China

**Keywords:** sustained-release membrane materials, physicochemical properties, analytic hierarchy, comprehensive evaluation

## Abstract

In this paper, the optimal analytic hierarchy process was used to establish a comprehensive evaluation model for the physicochemical properties of composite sustained-release membrane materials based on water absorption (*XS*), water permeability (*TS*), tensile strength (*KL*), elongation at break (*DSL*), fertilizer permeability (*TF*), and viscosity (*ND*), and the optimal ratio parameters of membrane material were determined. Analytic hierarchy process (AHP) combined with correlation analysis was used to construct the judgment matrix of physicochemical properties, which passed the consistency test, and to determine the weight and ranking of each index: *TF* (0.6144) > *XS* (0.1773) > *KL* (0.1561) > *ND* (0.1311) > *TS* (0.0775) > *DSL* (0.0520). The comprehensive scores of sustained-release membrane materials under different treatments were calculated based on normalized data samples and weights. It was determined that the percentage of each component in the best comprehensive performance of the slow-release membrane material was as follows: polyvinyl alcohol, polyvinylpyrrolidone, zeolite, and epoxy resin were 7.3%, 0.7%, 0.5%, and 2%, respectively.

## 1. Introduction

With the massive use of traditional fertilizers, environmental pollution, economic effects, food safety, and other problems are becoming increasingly obvious [1,2,3]. Biodegradable slow-release fertilizer can significantly improve the utilization rate of fertilizer, reduce environmental pollution, and meet the nutrient requirements of crops in the longer growth period [4,5,6]. However, slow-release fertilizer still has problems, such as low strength, strong hydrophilicity, and poor slow-release effect [7,8], so it is very important to optimize the type and composition ratio of membrane materials to solve these problems [9]. There are many types of membrane materials and complex proportions of components, which have different influences on the intensity and trend of each index, so it is difficult to optimize and evaluate the membrane materials. The method of a fuzzy comprehensive evaluation system based on multiple indices can provide an important theoretical tool for the optimization and preparation of membrane materials.

Previous studies mainly analyzed the fertility permeability (*TF*) [10], water absorption (*XS*) [11], tensile strength (*KL*) [12], elongation at break (*DSL*) [13], and other membrane properties responsive to various factors. For example, when PVDF powder was added into the mixture of DMF and acetone with the weight ratio of 9: 1, the *KL* of composite membrane reached the optimal value of 32.28 Mpa, and the *DSL* decreased to the lowest value of 26.21% [14].Adding glutaraldehyde at 0.3 mL brought the Young’s modulus of the membrane to an optimal value of 30.94 MPa, whereas the *DSL* was reduced to a lower level of 16.27% [15]. With 0.5 mL glycerol, the *DSL* of the membrane reached the best, and the *KL* reached the lowest level of 0.47 Mpa [16]. The mechanical properties of the membrane reached the optimal value of 20.75 MPa when the lees content was 15%, but the nitrogen release rate reached 18.55% after 24 h [17]. When the amount of Cu-MOF was 0.5 wt%, the contact angle of the composite membrane decreased from 75.27° to 58.05°, while the porosity of the composite membrane increased from 58.13% to 62.01% [18]. When 5% iodine was added into ethanol solution, the Young’s modulus and tensile strength of the composite membrane were decreased by 15.08% and 13.79%, respectively, while the elongation at break was increased by 118.18% [19]. This showed that the response strength and trend of each membrane index to the same factor are different, and the optimal treatment of each index lacks consistency; if the optimal evaluation method based on a single index could have subjective one-sidedness, it is necessary to adopt the multi-index objective comprehensive evaluation method [20].

The comprehensive evaluation method is an important step to reasonably determine the index weight and to obtain the evaluation result. Only by selecting appropriate, comprehensive evaluation methods for different problems can the evaluation results be accurate and scientific. Entropy weight method is a method to obtain information entropy and related weight according to the variation degree of information contained in each index [21]. It has been used in crop yield evaluation [22], machinery and equipment optimization [23], transportation development [24], etc. However, the weight obtained from the complete objectivity of entropy weight method may be inconsistent with the actual importance degree. The independence weight coefficient method was used to calculate the complex correlation coefficient to determine the weight by using the method of multiple regression analysis, which was applied to the weight evaluation calculation of water source index [25]. Although the independence weight coefficient method has strong objectivity, it can fully reflect the influence and effect of each index on the properties of membrane materials. However, in the process of weight assignment, some indicators with higher importance will be given lower weights. The analytic hierarchy process (AHP) is a comprehensive evaluation method, which can divide the elements related to decisions into several levels, such as objective, scheme, and criterion, so as to make a comprehensive analysis and give objective and reasonable optimal comprehensive evaluation results [26]. This method has been used in fruit crop evaluation [27,28], traffic safety construction [29,30], and corporate economic development [31,32], but there are few reports on the preparation process optimization of slow-release membrane materials. The traditional analytic hierarchy process is a subjective weighting method based on the experience and knowledge of experts, which lacks an objective basis in determining the weight of indicators, and strong subjective factors will affect the evaluation results [33,34]. In the establishment of weight, if the inherent laws and information of the original data cannot be analyzed and explored, the experimental results will also have a large error. In this paper, the optimal analytic hierarchy process was used to construct a pair comparison matrix according to the correlation coefficient between indexes and the evaluation criteria of the 1–9 scale method. Through a series of stable transfer matrices, it satisfies the consistency test and makes up for the problems of fuzziness, blindness, and subjectivity in the traditional analytic hierarchy process.

In this paper, the analytic hierarchy method was used to reasonably establish the weights of each index on the basis of the correlation analysis of six physical and chemical performance indices, including water absorption (*XS*), water permeability (*TS*), tensile strength (*KL*), elongation at break (*DSL*), fertilizer permeability (*TF*), and viscosity (*ND*) of slow-release membranes. Based on the subordinate function, a comprehensive evaluation system was constructed, and the optimal membrane material with comprehensive performance was chosen to provide a theoretical foundation for the objective comprehensive evaluation of the properties of slow-release membrane materials.

## 2. Materials and Methods

### 2.1. Data Source

The data in this study are derived from the preparation experiments of slow-release membrane materials under different ratios of water copolymer and zeolite. Four levels of water-based copolymer ratio A (PVA: PVP) were designed as A_1_–A_4_, with proportions of 8%: 0%, 7.3%: 0.7%, 6.6%: 1.4%, and 5.9%: 2.1%, respectively. Four levels of B(zeolite amount) were also designed as B_1_–B_4_, and the ratios in solution were 0%, 0.25%, 0.5%, and 1%, respectively. The amount of epoxy resin added was 14 g, equal to 2% in the solution. A two-factor four-level comprehensive experimental design with 16 groups was adopted. The determination of *XS*, *TS*, *KL*, *DSL*, *TF*, and *ND* proceeded through reference to research methods of predecessors [35,36,37,38,39].

### 2.2. Data Processing and Statistical Analysis

Microsoft Office 365 was used for data processing and table drawing. IBM SPSS Statistics 22 data analysis software was used to analyze the correlation of physical and chemical properties of membrane materials. Yaahp 10.3 was used to determine the index weight and to construct the comprehensive evaluation model of the membrane material.

### 2.3. Steps and Principles of Optimal AHP

The hierarchical analysis method, which was based on correlation analysis of the physicochemical properties of membrane materials, was used in this paper. The main principles and steps are as follows:

1. Build a hierarchical model: A hierarchy diagram was constructed based on *XS*, *TS*, *KL*, *DSL*, *TF*, and *ND*.
2. Construct the optimal judgment matrix: The degree of correlation among *XS*, *TS*, *KL*, *DSL*, *TF*, and *ND* was analyzed, and the paired comparison matrix was constructed by combining the evaluation criteria of the 1–9 scale method.
3. Hierarchical ranking and consistency check: The consistency of the judgment matrix was checked by calculating the CR value.
4. Calculate the comprehensive score of each index: The weight of each index was multiplied by the standardized value and then accumulated to obtain the comprehensive score of each treatment.

## 3. Results and Analysis

### 3.1. Statistical Analysis of Membrane Material Indices

**Figure 1:**
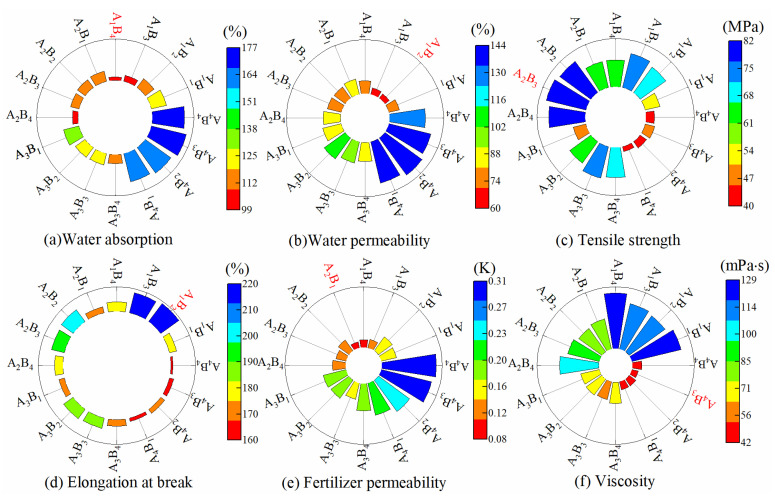
Parameters of physicochemical properties of membrane materials.

Figure 1 shows the index parameters of *XS*, *TS*, *KL*, *DSL*, *TF*, and *ND* of membrane materials under different water-based copolymer ratios and zeolite amounts. It can be seen from Figure 1 that in the condition of B_1_–B_4_, when A was decreased from A_1_ to A_4_, *XS*, *TS*, and *TF* increased by 51.6%, 101.1%, and 49.5%, while *DSL* and *ND* decreased by 15.7% and 61.9%, on average. *XS*, *TS*, and *TF* showed a positive response to the decrease of A, while *DSL* and *ND* showed a negative response. In the condition of B_1_–B_4_, when A was decreased from A_1_ to A_2_, *KL* increased by 15.4%, and when A was decreased from A_2_ to A_4_, *KL* decreased by 41.9%, on average. This finding showed that the decrease in A on *KL* was promoted first and then suppressed. In the condition of A_1_–A_4_, when B was increased from B_1_ to B_4_, *KL* and *DSL* were initially increased by 31.6% and 12.9%, and then decreased by 6.2% and 9.9%, on average. This finding revealed that the increases in B on *KL* and *DSL* were promoted first and then inhibited. Except for the A_4_ condition, *XS* was decreased by 15.5%, on average. *XS* presented a negative response to B increase, while the increase in B on *XS* was promoted first and then inhibited in the A_4_ condition. The *ND*, *TS*, and *TF* variations caused by B were 4.33%, 5.35%, 10.85%, respectively, suggesting that B increase had less effect on *ND*, *TS*, and *TF*. The slow-release membrane material with excellent comprehensive performance should have better water resistance, mechanical properties, slow-release property, and low viscosity [40,41,42]. Based on this principle, *XS*, *TS*, *KL*, *DSL*, *TF*, and *ND* reached the optimal values in A_1_B_4_, A_1_B_2_, A_2_B_3_, A_1_B_2_, A_2_B_1_, and A_4_B_3_ treatments, respectively. The above results indicated that the optimal treatment corresponding to each physicochemical property index of membrane materials is not consistent. If the optimal evaluation method based on a single index has subjective one-sidedness, the multi-index objective comprehensive evaluation method must be used. 

### 3.2. Construction of the Model Hierarchy

**Figure 2:**
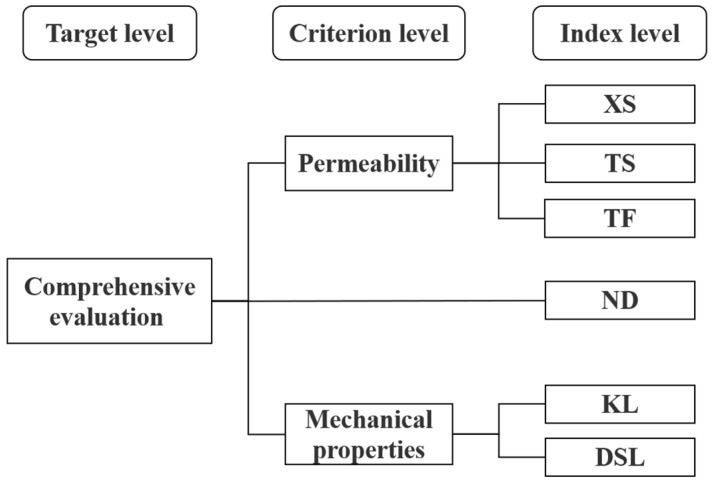
Decision tree for comprehensive evaluation of membrane materials.

Figure 2 shows the hierarchical index system can be generally divided into three levels: the top level, the middle level, and the bottom level. There was only one element in the top level, namely the target level. In this study, the target level was a comprehensive evaluation of physicochemical properties of membrane materials. The middle level was also called the criterion level, which in this study was divided into permeability and mechanical properties. The bottom level was also called the index level. According to the complexity and scale of the problem to be solved, the index level can be further divided. In this study, the index level was divided into six levels: *XS*, *TS*, *KL*, *DSL*, *TF*, and *ND*.

### 3.3. Establishment of Judgment Matrix

The correlation analysis of the six indices of the membrane is shown in Table 1. Table 1 shows that there was a certain degree of correlation among the different indicators. The correlation coefficient between *XS* and *TF* was 0.913, and there was a significant positive correlation between them (*p* < 0.01). *TS* was positively correlated with *TF* and *XS* (*p* < 0.01), and the correlation coefficients were 0.839 and 0.886, respectively. *XS* and *TS* were negatively correlated with *ND*, *KL*, and *DSL*, indicating that the higher the water absorption rate of the membrane material, the more serious the swelling inside the membrane material, and the mechanical properties of the membrane changed significantly [43].

Taking the overall optimization of the target level as the standard. In the hierarchical structure model of membrane material, it is necessary to make a pairwise comparison of the indices at the same level to establish the judgment matrix *A* = (*a_ij_*) *n* × *n*, and the judgment matrix must meet the following conditions: *a* > 0, $a_{ij}=\frac{1}{a_{ij}}$, (*i,j* = 1, 2, 3, …, *n*).

(1) $$A=\left(\begin{array}{ccc} a_{11} & \ldots & a_{1n}\\ \vdots & \ddots & \vdots \\ a_{m1} & \cdots & a_{mn} \end{array}\right)$$

The scale definition of judgment matrix is shown in Table 2.The pairwise comparison matrix was constructed based on the correlation analysis between different indices of membrane materials and the assignment standard of 1–9 scale method [44]. The importance degree of *TF* was regarded as 1 according to the important principle of fertilizer permeability of sustained-release membrane material. Table 1 shows that the order of correlation between other indices and *TF* is *XS* > *TS* > *ND* > *KL* > *DSL*. The higher correlation between two factors, the closer importance of the two factors. The values were assigned according to the degree of correlation between other indices and *TF*. The constructed judgment matrix is shown in Table 3 and the rest of the judgment matrices, which are shown in Table 4 and Table 5, are constructed in the same way.

### 3.4. Consistency Check of Judgment Matrix

In order to ensure the rationality of the weight distribution of each index in the comprehensive evaluation system, it is necessary to check the consistency of the judgment matrix of each level. First, the maximum characteristic root of the judgment matrix was calculated, and then the maximum characteristic root was used to calculate the *CI* value, which was used as the consistency index for consistency checking. The *CI* value was further used to obtain the *CR* value of the consistency index. Generally, the smaller the *CR* value, the more reasonable the judgment matrix and the higher the consistency. The procedure is as follows:

(2) $$\lambda_{\max}^{}=\frac{1}{n}\sum_{i=1}^{n}\frac{(X\omega {)}_{i}^{}}{\omega_{i}^{}}$$

In Formula (2): λ_max_ is the maximum characteristic root of the judgment matrix, *ω* is the weight vector, *ω_i_* is the weight of the ith evaluation index, and *n* is the number of evaluation index.

(3) $$CI=\frac{\lambda_{\max}^{}-n}{n-1}$$

In Formula (3): *CI* is the consistency index, *λ*_max_ is the maximum characteristic root of the judgment matrix, and *n* is the number of evaluation indices.

(4) $$CR=\frac{CI}{RI}$$

In Formula (4): *CR* is the random consistency ratio, *CI* is the consistency index, and *RI* is the consistency index of matrix average.

When the *CR* value is less than 0.10, the judgment matrix meets the consistency test; if it is greater than 0.10, the matrix does not have consistency, and the matrix should be adjusted and analyzed until it meets the consistency requirements. After calculation, the *CR* values of the judgment matrices corresponding to Table 3, Table 4 and Table 5 are 0.052, 0.071 and 0, respectively, which have good consistency, and all pass the consistency test.

### 3.5. Establishment of Index Weight

After calculation, the weight distribution of each evaluation index is shown in Table 6. As can be seen from Table 6, in the evaluation criterion level, the weight of permeability is the largest, reaching 0.661. The weights of mechanical properties and *ND* are 0.208 and 0.131, respectively. Permeability has the greatest influence on the properties of membrane materials, followed by mechanical properties, and *ND* has the least influence. The weight of *TF* was 0.614, which accounted for the largest weight in permeability, and the ratio of *TF* in the total weight of the six indices was also the largest, reaching 0.406. This shows that *TF* is an important index to evaluate the comprehensive performance of slow-release membrane materials [45,46]. Among the mechanical properties, the weight of *KL* is 0.750, which shows that *KL* is also important for the properties of membrane materials. The correlation analysis showed that the physical-chemical properties of membrane materials were independent and complex. The weights of all indices were in the following order: *TF* > *XS* > *KL* > *ND* > *TS* > *DSL*.

### 3.6. Comprehensive Evaluation of Membrane Materials

Before the comprehensive score calculation, the index data were normalized by the membership function method. Among the indices of membrane materials, *XS*, *TS*, *TF*, and *ND* were the minimum attributes, which were calculated by the following formula:

(5) $$U=(X_{\max}^{}-X)/(X_{\max}^{}-X_{\min}^{})$$

*KL* and *DSL* are maximum attributes, which can be calculated by the following formula:

(6) $$U=(X-X_{\min}^{})/(X_{\max}^{}-X_{\min}^{})$$

**Figure 3:**
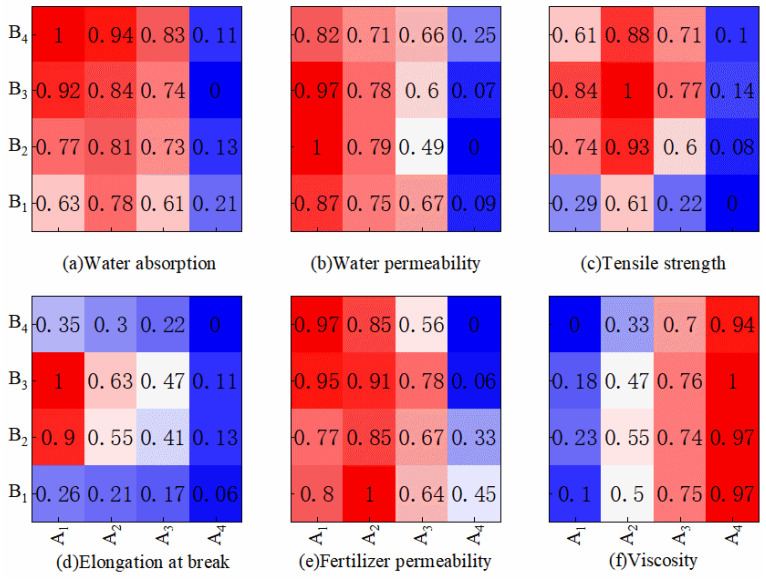
Membership function value of each treatment.

In Formulas (5) and (6): U represents the membership function value of the index, *X*_min_ indicates the minimum value of the index, and *X*_max_ indicates the maximum value of the indicator. Figure 3 shows the membership function values of each treatment.

The comprehensive score value of each treatment can be calculated by the following formula:

(7) $$W_{i}^{}=X_{1i}^{}Q_{1}^{}+X_{2i}^{}Q_{2}^{}+X_{3i}^{}Q_{3}^{}+X_{4i}^{}Q_{4}^{}+X_{5i}^{}Q_{5}^{}+X_{6i}Q_{6}$$

**Figure 4:**
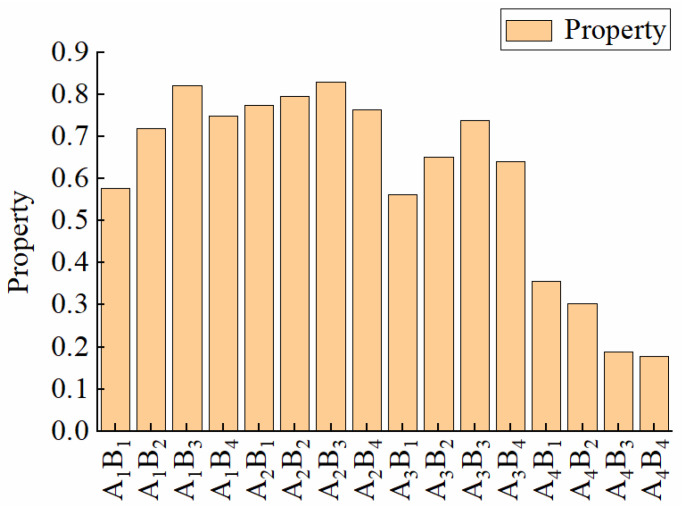
Comprehensive score values of different treatments.

In Formula (7): *W_i_* represents the composite score of the ith treatment (*i* = 1, 2, 3, …, 16), *X_li_* is the membership function value of the lth index of the ith treatment (*l* = 1, 2, 3, …, 6), and *Q_l_* indicates the weight of each index. The comprehensive score values of different treatments are shown in Figure 4.

## 4. Discussion

The results of this study showed that *XS* was negatively correlated with *KL* and *DSL*. You et al. [47] concluded that *XS* decreased with the increase of *KL* and *DSL*, which was consistent with the results of this paper. The entry of water molecules causes the membrane material to swell, which increases molecular distance and decreases the crosslinking degree of crosslinking groups, weakening the membrane material’s mechanical properties. The proper selection of indicators is especially important when evaluating membrane materials. If the previous evaluation method based on the *TF* index was used [48], the optimal treatment of membrane material was selected as A_2_B_1_ in this paper. The comprehensive evaluation method based on hierarchical analysis of six indices, including *XS*, *TS*, *KL*, *DSL*, *TF*, and *ND*, was proposed in this paper, the responses among the indices were considered, and the optimal membrane material was selected as A_2_B_3_ treatment. Data sample analysis showed that there was a difference of about 3% between A_2_B_1_ and A_2_B_3_ treatment in *XS*, *TS*, *ND*, and *TF*, indicating that the two treatments have similar performance. However, in terms of *KL* and *DSL*, A_2_B_3_ treatment was better than A_2_B_1_ treatment by 16.23 Mpa and 20.14%, respectively, showing better comprehensive performance. As a result, the results obtained in this paper by using multi-index comprehensive evaluation were more objective and reasonable.

In the comprehensive evaluation system, the establishment of reasonable weight is very important to solve the decision problem, and it is also a key factor for the accuracy of evaluation [49]. The optimized analytic hierarchy process was used to determine that the weight of permeability was 0.661, which occupied the largest proportion in the evaluation criterion level. The weight coefficients of *TF* and *XS* in the evaluation index level were 0.406 and 0.177, respectively, which showed that *TF* and *XS* were important indices to evaluate the properties of membrane materials [50]. The basis of a comprehensive evaluation is the reasonable establishment of weight, but the interrelationship between different indicators cannot be ignored. Pan [51] subjectively evaluated the performance indices of the prepared water-soluble membrane materials but ignored the establishment of the weight when evaluating and selecting the optimal membrane materials, and the test results showed a certain degree of subjectivity. In this paper, the importance of the evaluation criterion level and the correlation of the index level were considered in the comprehensive evaluation. After the reasonable establishment of weights, the score obtained by the membership function normalization was more objective and reasonable.

The reasonable choice of evaluation method is also crucial for whether the decision problem can be solved [52]. The index weights established based on the entropy weight method and the independence weight coefficient method are shown in Table 7 and Table 8, respectively. It can be seen from Table 7 that the weight of all indices established by the entropy value method were in the order: *DSL* > *KL* > *ND* > *TS* > *XS* > *TF*. It can be seen from Table 8 that the weight of all indices established by the independence weight coefficient method were in the order: *DSL* > *ND* > *TF* > *KL* > *TS* > *XS*. The entropy weight method determined that the index with the largest weight was *DSL*, which deviated from the more important goal of the fertilizer permeability of the slow-release membrane material. Therefore, the entropy weight method was not reasonable when determining the index weight in this paper. Following a comprehensive evaluation of membrane materials using the entropy weight method, it was determined that A_1_B_3_ was the best membrane treatment in terms of overall performance. The physical and chemical properties, such as *XS*, *TS*, and *KL*, of the A_1_B_3_ treatment were close to those of the A_2_B_3_ treatment, but the *ND* was far worse than that of the A_2_B_3_ treatment. Therefore, the entropy weight method was not reasonable in the weight establishment and comprehensive score calculation of the sustained-release membrane material indices in this paper. The index with the largest weight established by the independence weight coefficient method was *DSL*, which was the same as the maximum weight index established by the entropy weight method. However, the weight of *TF* in the independence weight coefficient method was higher than that in the entropy weight method, which made the independence weight coefficient method perform more reasonably. In this paper, the independence weight coefficient method was adopted for comprehensive evaluation of membrane materials, and it was concluded that the optimal membrane treatment was A_1_B_3_, which had the same defect as the entropy weight method. Both the entropy weight method and the independence weight coefficient method have irrationality in the weight distribution of the membrane material index, and the optimal treatment calculated by the method performs poorly in the *ND* index. In this paper, the optimal analytic hierarchy process was used to construct a judgment matrix combining the correlation between indices in the comprehensive evaluation, and the weight and optimal treatment obtained by combining subjective and objective methods were more reasonable.

In order to further explore the differences between the results of the three evaluation methods, the comprehensive scores of the three evaluation methods were jointly analyzed by Spearman correlation analysis. Table 9 shows the correlation analysis results of different evaluation methods. The optimal analytic hierarchy process was significantly correlated with entropy weight method and independence weight coefficient method. This showed that the evaluation results of optimal analytic hierarchy were in good agreement with entropy weight method and independence weight coefficient method.

## 5. Conclusions

An improved analytic hierarchy process was used to construct a comprehensive evaluation system for slow-release membrane materials based on six indices, including *XS*, *TS*, *KL*, *DSL*, *TF*, and *ND*. The weights of all indices were in the order: *TF* > *ND* > *TS* > *KL* > *XL* > *DSL*. The comprehensive scores of membrane materials under different conditions were clarified, and the optimal membrane material treatment was A_2_B_3_.

## Figures and Tables

**Table 1:** Correlation analysis of membrane material indices.

Index	XS	TS	ND	KL	DSL	TF
XS	1	0.886 **	−0.770 **	−0.865 **	−0.611 *	0.913 **
TS	0.886 **	1	−0.865 **	−0.755 **	−0.713 **	0.839 **
ND	−0.770 **	−0.865 **	1	0.571 *	0.593 *	−0.783 **
KL	−0.865 **	−0.755 **	0.571 *	1	0.720 **	−0.757 **
DSL	−0.611 **	−0.713 **	0.593 *	0.720 **	1	−0.594 *
TF	0.913 **	0.839 **	−0.783 **	−0.757 **	−0.594 *	1

** indicates that the correlation between factors is highly significant (*p* < 0.01), * indicates that the correlation between factors is significant (*p* < 0.05).

**Table 2:** Judgment matrix scale definition.

Scale	Implication
1	The two factors are of equal importance
3	The former is slightly more important than the latter
5	The former is more important than the latter
7	The former is strongly important compared to the latter
9	The former is extremely important compared to the latter
2, 4, 6, 8	The judgment is of intermediate value
Reciprocal	If the ratio of the importance of factor *i* to factor *j* is *a_ij_*, then the ratio of factor *j* to the importance of factor *i* is *a_ij_* = 1/*a_ij_*

**Table 3:** Pairwise comparison matrix of index level.

P_1_	XS	TS	TF
XS	1	3	1/3
TS	1/3	1	1/4
TF	3	4	1

**Table 4:** Pairwise comparison matrix of criterion level.

P_2_	Mechanical Properties	Permeability	ND
Mechanical properties	1	1/4	2
Permeability	4	1	4
ND	1/2	1/4	1

**Table 5:** Pairwise comparison matrix of index level.

P_3_	KL	DSL
KL	1	3
DSL	1/3	1

**Table 6:** Weight distribution of different indices.

Evaluation Criterion Level	Weight	Evaluation Index Level	Weight	Total Weight
Permeability	0.661	TF	0.614	0.406
		TS	0.117	0.078
		XS	0.268	0.177
Mechanical properties	0.208	KL	0.750	0.156
		DSL	0.250	0.052
ND	0.131	ND	0.131	0.131

**Table 7:** Weight of each index based on entropy method.

Index	KL	DSL	TF	TS	ND	XS
Entropy value	0.912	0.892	0.946	0.931	0.929	0.936
Weight	0.193	0.238	0.119	0.153	0.156	0.141

**Table 8:** Weight of each index based on independence weight coefficient method.

Index	KL	DSL	TF	TS	ND	XS
Multiple correlation coefficient	0.925	0.827	0.924	0.945	0.891	0.969
Weight	0.1642	0.1836	0.1644	0.1608	0.1705	0.1566

**Table 9:** Correlation analysis among different evaluation methods.

Evaluation Method	Optimal Analytic Hierarchy Process	Entropy Weight Method	Independence Weight Coefficient Method
Optimal analytic hierarchy process	1		
Entropy weight method	0.952 **	1	
Independence weight coefficient method	0.973 **	0.995 **	1

** indicates that the correlation between factors is highly significant (*p* < 0.01).

## Data Availability

Not applicable.
